# Radiotherapy of early‐stage breast cancer

**DOI:** 10.1002/pro6.1183

**Published:** 2023-01-29

**Authors:** Cedric X. Yu

**Affiliations:** ^1^ Radiation Oncology University of Maryland School of Medicine Baltimore Maryland USA; ^2^ Xcision Medical Systems Columbia Maryland USA

**Keywords:** early‐stage breast cancer, partial breast irradiation, preoperative RT, radiation therapy, whole‐breast irradiation

## Abstract

Breast cancer is the most prevalent disease for women. With advances in breast cancer screening, most breast cancers are now diagnosed in the early stages. With knowledge of different subtypes and their behavior, breast cancer treatment has become more individualized. Radiation therapy as one of the mainstays of breast cancer treatment has also been evolving. This review attempts to provide a summary of the most influential clinical studies that have driven the technological advances in radiation therapy for early‐stage breast cancer.

## Graphical abstract

Breast cancer is the most prevalent disease for women. With the advances in breast cancer screening, most breast cancers are now diagnosed in the early stage. With the knowledge of different subtypes and their behavior, breast cancer treatment has become more individualized. Radiation therapy as one of the mainstays of breast cancer treatment has also been evolving. This review attempts to provide a summary of the most influential clinical studies that have driven the technological advances in radiation therapy for early stage breast cancer.

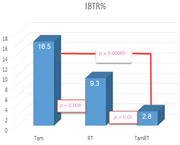

## 1 WHOLE‐BREAST IRRADIATION

### 1.1 Breast‐conserving treatment

In 1985, Fisher et al.^1^ published a landmark study of 1843 women that indicated that lumpectomy plus postoperative radiation therapy is appropriate for patients with stage I or II breast cancer with negative margins. In the study, patients were randomized to total mastectomy, lumpectomy alone, or lumpectomy followed by radiation therapy. It was shown that for these early‐stage breast cancers, lumpectomy was equivalent to mastectomy in terms of disease‐free survival and overall survival (OS). Furthermore, the addition of postoperative radiation therapy further improves both local control and survival.

Subsequently, multiple phase III clinical trials across different continents enrolling tens of thousands of women have confirmed Fisher's pioneering results. A summary of these trials is provided by the Early Breast Cancer Trialists’ Collaborative Group (EBCTCG).^2^ By analyzing 42,000 patients in 78 clinical trials, the results show that adding radiation therapy to breast conserving surgery (BCS) not only reduces absolute ipsilateral breast tumor recurrence (IBTR) by over 19%, such reduction also translated to advantages in both disease‐specific survival (DSS) and OS by more than 5%. They concluded that for every four IBTR prevented, one life will be saved.

Based on the EBCTCG analyses, Punglia et al.^3^ demonstrated that breast cancer is neither purely systemic nor purely local disease, but a spectrum between the two extreme views. Local recurrence can lead to remote metastasis, reducing a patient's chance of long‐term survival.

Low recurrence rates and relative noninvasiveness are thus the rationale for lumpectomy plus radiation therapy in place of mastectomy for eligible patients. Currently, in western Europe, 60–80% of newly diagnosed cancers receive breast‐conservation therapy (BCT).^4^

### 1.2 Role of radiotherapy post tamoxifen

A full review on the radiotherapy of breast cancer is not possible without giving due credit to tamoxifen. Synthesized in the 1960s, tamoxifen failed its original purpose as a candidate contraceptive and later became the gold standard for the endocrine treatment of all stages of estrogen‐receptor (ER)‐positive breast cancer.^5^, ^6^ Tamoxifen binds to ER, thereby blocking the estrogen and cutting off the fuels to tumor growth. Its precision targeting of ER‐positive breast cancer cells makes tamoxifen the world's first “targeted agent.”

The success of tamoxifen in both the treatment of breast cancer and prevention of tumor recurrence led to numerous clinical trials that challenge the role of radiotherapy. The most influential phase III trials are summarized below.

#### 1.2.1 NSABP B21^7^

**FIGURE 1:**
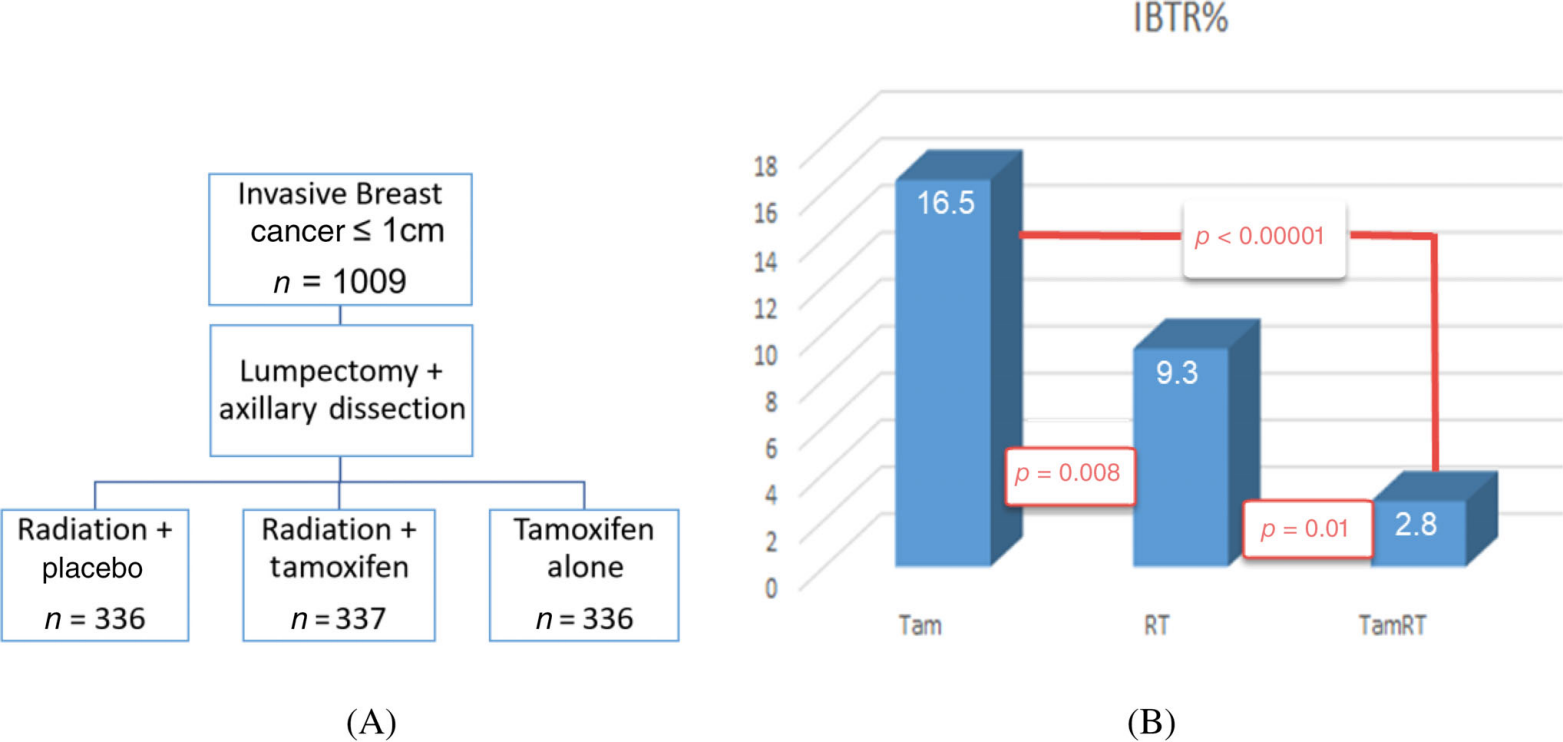
(A) B21 study illustration and (B) 8‐year cumulative IBTR results

The design of the NSABP B‐21 study is illustrated in Figure 1A. It was designed to examine the effectiveness of tamoxifen and postoperative radiation in preventing IBTR for very early‐stage invasive breast cancer. A total of 1009 patients with tumor size ≤1 cm and negative nodes were randomly assigned to radiation plus placebo, radiation plus tamoxifen, and tamoxifen alone after lumpectomy and axillary node dissection.

The results of the 8‐year follow‐up are illustrated in Figure 1B. It showed that radiation plus placebo was more effective than tamoxifen alone (9.3% vs. 16.5%, *p* = 0.008) and adding radiation to tamoxifen reduced IBTR to 2.8%. Therefore, even for very small breast cancers with negative nodes, radiation is more effective in controlling IBTR than tamoxifen alone. The combination of both tamoxifen and radiation is more effective than either modality alone.

#### 1.2.2 CALGB 9343^8^

From the early days of breast‐conserving therapy, for decades postoperative radiation therapy predominantly consisted of 5 weeks of daily irradiation of the entire breast. Because of the protracted course of treatment, there was a strong motivation for omitting radiation therapy after breast‐conserving surgery. CALGB 9343 was to answer the question of whether omitting radiation is possible for women aged ≥70 years with early‐stage breast cancer. A total of 636 women with clinical stage I (T1N0M0 according to TNM classification) ER‐positive breast cancer were equally randomized to receive either tamoxifen alone or tamoxifen plus radiation after lumpectomy.

At 10.5 years, patients who received both tamoxifen and radiation had a IBTR of 2% (95% confidence interval [CI] 1–4%) compared with 9% of those receiving tamoxifen alone (95% CI 7–15%). There were no statistically significant differences in terms of time to mastectomy, time to distant metastasis, DSS, or OS between the two groups.

Although there were several folds of difference in IBTR, the authors concluded that radiation added no significant benefit in terms of survival and that tamoxifen alone was a reasonable option for women aged ≥70 years with ER‐positive early‐stage breast cancer. One contributing factor to this conclusion was the fact that these were older patients and the total number was not high enough to provide the statistical power to distinguish small but clinically meaningful differences in OS.

#### 1.2.3 PRIME II study^9^

This was a multicenter European study to determine whether there is a benefit to adjuvant radiation therapy after breast‐conserving surgery and tamoxifen in women aged ≥65 years with early‐stage breast cancer. A total of 1326 women were enrolled, with 658 randomly assigned to tamoxifen plus radiation (TamRT) and 668 to tamoxifen (Tam) alone. The first report was published with a 5‐year follow‐up.^9^ The rates of IBTR were 1.3% for TamRT and 4.1% for Tam alone. No differences in regional recurrence, distant metastases, contralateral breast cancers, new breast cancers, or OS were noted between the two groups. The authors concluded that the absolute difference of IBTR was low enough to omit radiotherapy for these low‐risk patients.

The authors reported the updated 10‐year results at the 2021 San Antonio Breast Cancer Symposium.^10^ With a median follow‐up of 7.3 years, the 10‐year rates of IBTR were 0.9% (95% CI 0.1, 1.6%) for TamRT and 9.8% (95% CI 6.5, 13.2%) for Tam alone (*p* < 0.0001). Differences were also found for regional recurrence (2.3% Tam vs. 0.5% TamRT, *p* = 0.014), distant metastasis (3.6% Tam vs. 1.4% TamRT), and mastectomies at 5 years (1.8% Tam vs. 0.3%TamRT). Again, no differences were found in DSS and OS. The authors again concluded that it was a reasonable option to omit postoperative radiation therapy for these early‐stage, hormone‐receptor‐positive breast cancer patients because there was no statistically significant difference in DSS and OS.

Through all these clinical trials aimed to omit radiotherapy, it was evident that radiotherapy could significantly reduce IBTR, even for the most favorable patient groups, but the differences in IBTR did not translate to statistically significant survival advantage. However, if one looks the date carefully, the 9% reduction of IBTR did translate to a reduction of approximately 2% in regional recurrence, in distant metastasis, and in the rate of salvage mastectomies, which roughly coincided with the “rule of thumb” of one life saved for every four IBTRs prevented. Nevertheless, these conclusions make protracted and invasive postoperative radiation therapy undesirable. On the other hand, if radiotherapy can be delivered in one or a few noninvasive treatments, these obvious benefits in IBTR and the reduced need for salvage mastectomy would not be ignored.

### 1.3 Progression of WBI techniques for BCT

#### 1.3.1 Conventionally fractionated WBI

Conventionally fractionated whole‐breast irradiation (CF‐WBI) is commonly delivered as 1.8–2.0 Gy per fraction for 25–28 fractions (total dose of 45–50 Gy) with or without a boost to the lumpectomy cavity. The boost is indicated for patients with unfavorable risk factors and typically 2 Gy per fraction for five to eight fractions (total dose of 10–16 Gy). CF‐WBI begins approximately 1 month after surgery and lasts for 5–7 weeks. WBI is delivered using a linear accelerator, and radiation beams direct tangentially to the chest wall with the patient usually in the supine orientation. Three‐dimensional conformal radiotherapy (3D‐CRT) or intensity modulated radiotherapy (IMRT) techniques are used. In either case, techniques are used to ensure uniform dose coverage of the whole breast while minimizing the dose to the thoracic organs (the heart and lungs). Prone techniques are also used, with the treated breast hanging freely and thus pulling the targeted breast tissue away from the chest wall and thoracic organs, thereby reducing doses to the heart and lungs.

#### 1.3.2 Hypo‐fractionated WBI

The use of larger doses per fraction over fewer fractions is known as hypo‐fractionation.

Multiple hypo‐fractionation trials were conducted in UK to determine if it is as safe and effective to deliver larger fractional dose over fewer treatments (HF‐WBI) as it is to deliver the conventional 50 Gy in 25 fractions (CF‐WBI). The UK START pilot trial assessed two doses (39 and 42.9 Gy) of a 13‐fraction regimen delivered over 5 weeks.^11^ The START‐A trial included two 13‐fraction regimens (39 and 41.6 Gy) and 5‐year results for local tumor control and late toxicity were found to be noninferior to CF‐WBI.^12^ The START‐B trial compared 40 Gy in 15 fractions over 3 weeks (used in the UK and Canada for decades) to CF‐WBI. Five‐year results again suggest that hypofractionation is noninferior to conventional fractionation in terms of both IBTR and late toxicity.^13^ The START results have subsequently formed the UK National Institute for Health and Care Excellence (NICE) and American Society for Radiation Oncology (ASTRO) guidelines for breast radiotherapy fractionation. Currently, the most widely used fractionation schedule in the UK is 40 Gy in 15 fractions over 3 weeks, as used in START‐B.^14^ Similar HF‐WBI trials were also conducted in Canada,^15^, ^16^ China,^17^ and Denmark.^18^ The dose/fractionation schemes of these trials are shown in Table 1. These trials all resulted in similar efficacy as CF‐WBI and some with significantly less moderate to severe breast induration, edema, and telangiectasia.

**TABLE 1:** Phase III trials comparing conventional fractionation with moderate hypofractionation

Trial	Trial dates	Dose/fractionation	Key findings
START Pilot^11^ (United Kingdom)	1986–1998	42.9 Gy in 13 fractions 39 Gy in 13 fractions 50 Gy in 25 fractions	Hypofractionation is noninferior in both IBTR and toxicities
START A^12^ (United Kingdom)	1998–2002	41.6 Gy in 13 fractions 39 Gy in 13 fractions 50 Gy in 25 fractions	Hypofractionation is noninferior in both IBTR and toxicities
START B^13^, ^14^ (United Kingdom)	1999–2001	40 Gy in 15 fractions 50 Gy in 25 fractions	Hypofractionation is noninferior in both IBTR and toxicities
Canadian trial^15^, ^16^ (Canada)	1993–1996	42.5 Gy in 16 fractions 50 Gy in 25 fractions	Hypofractionation is noninferior in both IBTR and toxicities
Chinese trial^17^	1993–1996	43.5 Gy in 15 fractions + tumor bed boost of 8.7 Gy in three fractions 50 Gy in 25 fractions + tumor bed boost of 10 Gy in five fractions	Hypofractionation is noninferior for high‐risk postmastectomy patients
DBCG trial^18^ (Denmark)	2009–2014	40 Gy in 15 fractions 50 Gy in 25 fractions	Hypofractionation does not increase the occurrence of breast induration at 3 years

Further reducing the number of fractions, the UK FAST trial evaluated the use of five fractions delivered once per week (28.5 or 30 Gy total). This was compared to CF‐WBI of 2 Gy fractions over 5 weeks. In terms of cosmetic outcome, the 28.5 Gy schedule showed comparable results to CF‐WBI and better results than the 30 Gy schedule.^19^, ^20^ Similarly, a study which was carried out in Turin, Italy delivering 5 weekly fractions to a total dose of 30–32.5 Gy showed excellent local control and cosmesis.^21^

In the UK FAST‐forward trial, five fractions were delivered over just 1 week to a total dose of 26 or 27 Gy. This was compared to HF‐WBI of 40 Gy in 15 fractions (control). A total of 4096 early‐stage breast cancer patients aged 50 or older were enrolled, evenly randomized to the three groups. Noninferiority was judged with a 1.6% margin with a critical hazard ratio (HR) of 1.81. Initial results showed that as compared to 40 Gy in 15 fractions, the 5‐day schedules led to a significant reduction of acute skin toxicity (13.6% for 40 Gy/15F, 9.8% for 27 Gy/5F, 5.8% for 26 Gy/5F).^22^ The investigators later reported the 5‐year results in the *Lancet*.^23^ Both five‐fraction schedules were not inferior to 40 Gy in 15 fractions in terms of IBTR (5‐year cumulative incidence of IBTR was 1.7% [95% CI 1.2–2.6] for the 27 Gy group, 1.4% [0.9–2.2] for the 26 Gy group, and 2.1% [1.4–3.1] for the 40 Gy group). These results have led to the wide adoption of HF‐WBI all over the world. These two trials with aggressive hypofractionation schedules are summarized in Table 2.

**TABLE 2:** Phase III trials of aggressive hypofractionation schemes

Trial	Trial dates	Dose/fractionation	Key findings
FAST^19^, ^20^ (United Kingdom)	2004–2007	28.5 Gy in five fractions once weekly 30 Gy in five fractions once weekly 50 Gy in 25 fractions	28.5 Gy in five once‐weekly fractions is noninferior to 50 Gy in 25 fractions in both local control and toxicity
FAST‐Forward^22^, ^23^ (United Kingdom)	2011–2014	26 Gy in five daily fractions 27 Gy in five daily fractions 40 Gy in 15 fractions	26 Gy in five daily fractions is noninferior to 40 Gy in 15 fractions for local control and safety

These results also confirm that the α/β ratio for late‐responding tissues is around 3 Gy and the insensitivity of early‐reacting self‐renewal tissues to fraction size as predicted earlier by Yarnold et al.^24^

## 2 PARTIAL BREAST IRRADIATION

Driven by the same desire of reducing the overall treatment duration as HF‐WBI, reducing the treatment volume with partial breast irradiation (PBI) has also emerged as a new technique. PBI using a variety of techniques is in widespread use both within and outside of clinical trials. Note that because the fractionation schemes used in PBI are shorter than those for WBI, treatment is accelerated and the technique is often referred to as accelerated partial breast irradiation (APBI). It is APBI that has led the application of stereotactic radiosurgery (SRS) and stereotactic body radiation therapy (SBRT) for early‐stage breast cancer. This section provides an overview of the techniques and major clinical studies on PBI.

### 2.1 Rationales for PBI

In 1985, by slicing frozen mastectomy specimens and carefully conducting pathological and microscopic analyses on all the slices, Holland et al.^25^ found that 63% of all invasive breast cancers contained tumorlets outside the main index lesion. By measuring the distances of these tumerlets to the index lesion, they found that the probability of the presence of a tumorlet decreased exponentially with the distance to the index lesion and more than 90% of these satellites were within a few centimeters. This characteristic explained not only the success of postoperative radiotherapy but also the clinical observation that most IBTRs after BCT were close to the original tumor site.^26^ These pathological and clinical observations are the key rationales for PBI.

In addition, since WBI incorporates the entire breast, overlying skin, lower axilla, and even small portions of the heart and lung in the treatment fields, this may introduce toxicity that can be avoided via restriction of the radiotherapy target volume to the tumor bed and its surrounding region. In particular, radiation‐induced lung injuries after WBI, such as pneumonitis, lung fibrosis, and pulmonary function test changes, are well documented.^27^ Darby et al.^28^ and van den Bogaard et al.^29^ each analyzed more than 900 patients with breast cancer treated with WBI radiotherapy, and found that the mean heart dose for WBI of the left breast ranged from 4.4 Gy with newer technology to 6.6 Gy with older technology. The risk of cardiac events increased by 7.4%/Gy and continued with time.

### 2.2 Techniques for partial breast irradiation

Several options exist for delivery of PBI, including brachytherapy using either intracavitary or interstitial approaches, external beam radiotherapy (EBRT), and intraoperative radiation therapy (IORT). These different modalities are likely to have certain tradeoffs regarding their effectiveness and toxicity profiles although these tradeoffs have yet to be completely described in the existing literature. When compared with WBI, all PBI strategies offer advantages of reduced treatment time and sparing of uninvolved tissue.^26^ A summary of the available PBI options is given in the following sections.

#### 2.2.1 Multicatheter interstitial brachytherapy

This is the APBI technique that has been utilized the longest and has the most extensive follow‐up. Typically, about 14 to 20 flexible catheters are placed through the breast tissues surrounding the lumpectomy, into which radioactive sources (Ir‐192) are inserted. The catheters are inserted at 1–1.5 cm intervals in several planes to ensure adequate coverage of the lumpectomy cavity plus margins and to minimize nonuniformity of dose. Optimum catheter placement requires skill and experience of practitioners, although modern planning techniques based on computer tomography (CT) help in this regard. Either low dose rate (LDR) or high dose rate (HDR) brachytherapy may be used. With LDR, many low‐activity sources are implanted for approximately 2–5 days while the patient is admitted as an inpatient. HDR uses a single high‐activity source placed sequentially at different points within the catheters. A typical fractionation scheme for HDR is a total dose of 34 Gy in 10 fractions delivered twice daily. HDR is thus more closely related in terms of radiobiology to fractionated external beam radiation therapy.

#### 2.2.2 Balloon and strut‐based intracavitary brachytherapy

Balloon‐based brachytherapy uses a saline‐filled balloon applicator placed within the lumpectomy cavity, with one or more high activity Ir‐192 sources or an electronic X‐ray source placed within catheters (lumens) inside the balloon. The balloon fills the cavity so that the tumor bed receives a uniform dose throughout its surface in contact with the balloon. However, the conformity of the balloon with the walls of the cavity is a critical factor in achieving dose uniformity. Furthermore, the traditional single‐lumen design, where the source can be positioned only along a catheter on the central axis of the balloon, limits the ability to customize the dose distribution and minimize dose to specific nearby tissues such as the skin, ribs, and organs at risk such as heart and lung. For this reason, multilumen designs have been introduced with additional catheters to offset a few millimeters from the balloon axis.

The balloon brachytherapy device with the longest history of clinical use is the single‐lumen MammoSite® system (Hologic). The MammoSite applicator can be placed into the lumpectomy cavity at the time of surgery or in a separate procedure after surgery. The Mammosite multilumen device uses three additional catheters (four in total), offset from the central catheter by 3 mm. This provides additional, potentially asymmetric, source positions and increases dosimetric flexibility compared with a single‐catheter approach. Hologic also markets the Contura® multilumen device, which has a slightly different configuration with four additional catheters (five in total), offset from the central catheter by 5 mm.

The Axxent® electronic brachytherapy (eB) system (Xoft) is a modified form of balloon‐based brachytherapy that uses an electronic 50 kV X‐ray source rather than an Ir‐192 source. The eB system's X‐rays are of significantly lower energy than Ir‐192 gamma rays and this causes differences in the pattern of dose deposition near to and further from the source. A study has shown that eB generally gives a lower dose to organs at risk due to the faster fall‐off of its lower energy radiation.^30^

An alternative to balloon brachytherapy is the Strut Adjusted Volume Implant (SAVI®; Cianna Medical). In a similar fashion to the multilumen balloon devices, this system uses multiple (six, eight, or 10) peripheral catheters that allow flexibility in the dose distribution. However instead of the sources being relatively central within a saline‐filled balloon that fills out the lumpectomy cavity, the strut design fills out the cavity with the catheters themselves. Thus, the source‐containing catheters are in contact with the cavity walls. This in principle allows more control of dose at different positions, for example to reduce dose at locations where the skin is close to the wall. A downside is reduced dose homogeneity compared to balloon devices and the risk of “hot” dose regions near the catheters due to the proximity of the sources to the cavity wall.

#### 2.2.3 External beam radiotherapy

EBRT includes photon beam 3D‐CRT, IMRT, and proton beam therapy. The key advantage of EBRT over brachytherapy techniques is that EBRT is noninvasive and the patient is not subjected to a second invasive surgical procedure or anesthesia. Furthermore, the treatment is weeks from surgery and the pathological results of the original tumor and the status of the resection margins are available (if margins are not free of cancer then WBI is likely to be indicated). With EBRT it is inherently easier than with brachytherapy to achieve a uniform dose throughout the desired treatment volume.

IMRT offers advantages over 3D‐CRT in terms of ability to conform the dose to the target volume and keep high doses away from selected normal tissues. Various forms of IMRT using multileaf collimators have become widely used due to this flexibility. Significantly, the UK IMPORT‐LOW (Intensity Modulated and Partial Organ Radiotherapy—Low risk patients) trial used a simple form of IMRT known as forward‐planned IMRT to deliver 40 Gy in 15 daily fractions.^31^ The favorable results of this technique relative to WBI have led to the technique becoming the recommended form of EBRT‐based PBI by the UK Royal College of Radiologists in *Postoperative radiotherapy for breast cancer: UK consensus statements*.^32^ The Danish Breast Cancer Group (DBCG) trial used the same technique and achieved similarly favorable results.^33^

Prone PBI has been investigated to reduce the effect of respiration and to increase the distance between the target volume and the heart and lungs.^34^ The technique is dependent on the use of a suitable immobilization device, either made in house or sourced commercially.^35^ The prominent New York University technique developed by Formenti et al. uses 30 Gy in five fractions delivered over 10 days.^34^

#### 2.2.4 Intraoperative radiation therapy

IORT delivers a single dose to the tumor bed immediate after lumpectomy. Mobile IORT systems using either low‐energy X‐ray beams or electron beams can be brought into the operating room. A special applicator is used to apply the radiation directly onto the tumor bed from within the lumpectomy cavity. This is a similar process to a single episode of HDR balloon brachytherapy (the Xoft Axxent electronic brachytherapy device can be used for IORT). IORT delivers the radiation dose directly to the surgical margins at the time of surgery. With electrons or low‐energy X‐rays, the treatment depths from the surgical cavity wall are generally short. The lower doses to skin and nontarget tissues could lower the toxicity to normal breast tissue and to the lung and heart. One potential disadvantage of IORT is that final pathology is not available at the time of irradiation, so information on whether surgical margins are clear of cancer is lacking. For this reason, subsequent EBRT may be required in addition to IORT for some patients.^36^ A further concern for low‐energy X‐ray IORT is that the rapid drop in dose away from the walls of the excision cavity may result in recurrences.^37^ Two large randomized studies have compared IORT to WBI. The TARGIT trial used a low‐energy X‐ray system (INTRABEAM; Carl Zeiss Surgical) delivering a dose of 20 Gy at the surface of the cavity wall but only 5–7 Gy at 1 cm depth away from the cavity wall.^38^ The ELIOT trial delivered 21 Gy using an electron beam system.^39^

### 2.3 Suitable population for partial breast irradiation

APBI has been tested in a limited number of trials with over 10,000 patients over the last 10 years. These trials show that in properly selected patients with breast cancer, APBI has provided outcomes that are similar to WBI. In general, the most suitable patients are those in an older age group with small, unifocal tumors that have clear surgical margins.

Both the American Society for Therapeutic Radiation Oncology (ASTRO) and the Groupe Européen de Curiethérapie‐European Society for Therapeutic Radiology and Oncology (GEC‐ESTRO) have made consensus statements on the guidelines and suitability criteria for patient selection^40^, ^41^ based on published clinical data.

The ASTRO guidelines state that suitable patients include those:

- 50 years of age or older with invasive ductal carcinoma measuring ≤2 cm (T1 disease) with negative margin widths of ≥2 mm, no lymph‐vascular space invasion (LVSI), ER positive, and BRCA gene negative, or
- With low/intermediate nuclear grade, screen‐detected ductal carcinoma in situ (DCIS) measuring ≤2.5 cm with negative margin width of ≥3 mm.

The GEC‐ESTRO breast cancer working group also made similar recommendations for patient suitability for APBI. The key differences between the GEC‐ESTRO and ASTRO recommendations are the inclusion of T2 disease and the explicit exclusion of lobular invasive breast cancer, the presence of an extensive intraductal component (EIC), and lympho‐vascular invasion (LVI).

### 2.4 Clinical trials of PBI and APBI

The UK IMPORT‐LOW trial^31^ is a phase III, noninferiority trial that compared two HF‐WBI regimens with two different doses and one PBI regimen. A total of 2018 women were enrolled and evenly assigned to these three cohorts. Of interest here is the comparison between the PBI and WBI using the same dose and fractionation. Using forward‐planned IMRT to deliver PBI of 40 Gy in 15 daily fractions achieved favorable results compared to WBI with the same dose and fractionation.^31^ Five‐year estimates of local relapse cumulative incidence were 1.1% of patients in the control WBI group and 0.5% in the PBI group (HR = 0.65, 95% CI 0.23–1.84), thus fulfilling the hypothesis of noninferiority of PBI. Patients in the PBI group also had no breast edema and significantly lower incidence of breast shrinkage.

The main characteristics of six major phase III randomized controlled trials using different types of APBI techniques are summarized in Table 3.

**TABLE 3:** Major phase III randomized controlled trials using different types of APBI techniques

Trial	PBI technique	PBI fractionation	Number of patients
NSABP B39/RTOG 0413 (USA)	3D‐CRT/IMRT Interstitial/Intracavitary brachytherapy	38.5 Gy in 10 fractions twice daily over 5–8 days 34 Gy in 10 fractions	4216
RAPID (Canada)	3D‐CRT	38.5 Gy in 10 fractions twice daily over 5–8 days	2135
GEC‐ESTRO (European)	HDR multicatheter brachytherapy	30.3–32.0 Gy in seven or eight fractions	1184
Florence (Italy)	IMRT	30 Gy in five fractions over 5 days	520
TARGIT (UK, Australia, Europe, USA)	IORT (X‐rays)	21 Gy in one fraction	3451
ELIOT (Italy)	IORT (electrons)	20 Gy in one fraction	1305

Abbreviations: 3D‐CRT, three‐dimensional conformal radiotherapy; HDR, high dose rate; IMRT, intensity modulated radiotherapy; IORT, intraoperative radiation therapy.

#### 2.4.1 NSABP B39 /RTOG 0413^42^, ^43^

Patients with breast cancers (tumor size ≤3 cm, including all histologies and multifocal breast cancers) who had conducted lumpectomy with negative surgical margins were randomly assigned (1:1) to receive either CF‐WBI with or without a supplemental boost to the tumor bed or APBI using EBRT, or intracavitary or interstitial brachytherapy. APBI was delivered as 34 Gy of brachytherapy or 38.5 Gy of EBRT in 10 fractions, over 5 treatment days within an 8‐day period. The absolute difference in 10‐year rate of IBTR between APBI and WBI was 0.7% (4.6% [95% CI 3.7–5.7] in the APBI group vs. 3.9% [3.1–5.0] in the WBI group). Due to the inclusion of higher risk patients and the trial design, the trial was unable to demonstrate equivalence between APBI and WBI.^43^

#### 2.4.2 The OCOG‐RAPID trial^44^

The RAPID trial compared APBI using only EBRT with the same dose fractionation as NSABP B39 (38.5 Gy/10 fractions twice daily [at least 6 h apart] using EBRT and WBI delivered with conventional fractionation). In total, 2135 patients were randomized (1:1) to the two cohorts. The absolute difference in 8‐year cumulative IBTR was 0.2% (3% after APBI or 2.8% after WBI, HR 1.27, 90%CI 0.84–1.91), achieving equivalence in terms of local control. However, it was found that APBI using EBRT was associated with a higher incidence of late toxicity than CF‐WBI (32% vs. 13% grade 2 or higher) and a higher number of fair or poor cosmetic outcomes evaluated by both the study nurses or by the patients themselves. Poor to fair cosmetic results ranged from 31% by patients to 36% by nurses at 7 years.

#### 2.4.3 GEC‐ESTRO interstitial APBI trial^45^

While the Canadian RAPID trial compared APBI using EBRT with CF‐WBI, this GEC‐ESTRO phase III trial compared APBI using multicatheter interstitial brachytherapy with CF‐WBI. A total of 1184 patients with low‐risk invasive breast cancer or DCIS were randomly assigned to either APBI using brachytherapy or CF‐WBI after breast‐conserving surgery. The absolute difference in IBTR at 5 years was 0.52% (95% CI 0.72–1.75, *p* = 0.42). APBI showed a slight trend of lower toxicity with 5‐year risk of grade 2–3 late side effects to the skin being 3.2% with APBI vs. 5.7% with CF‐WBI (*p* = 0.08), and both with very low 5‐year risk of grade 2–3 subcutaneous fibrosis.^46^ Therefore, the trial established equivalency between postoperative APBI using multicatheter brachytherapy and postoperative CF‐WBI for patients with early breast cancer in terms of local control, disease‐free survival, and OS.

#### 2.4.4 The University of Florence APBI trial^47^, ^48^

The University of Florence APBI trial established equivalency by comparing APBI that used EBRT delivering 30 Gy in five daily fractions with CF‐WBI after breast‐conserving surgery. In total, 520 low‐risk patients were randomly assigned to the two arms (WBI 260, APBI 260). The median follow‐up period was 10.7 years. The absolute difference in 10‐year risk of IBTR was 1.2% (2.5% with CT‐WBI and 3.7% with APBI). Both disease‐specific survival and OS at 10 years were similar. Contrary to the OCOG‐RAPID trial, the APBI arm showed significantly less acute toxicity (*p* < 0.0001), late toxicity (*p* < 0.0001), and improved cosmetic outcome as evaluated by both physicians (*p* < 0.0001) and patients (*p* < 0.0001).

#### 2.4.5 TARGIT trial^49^

The TARGIT trial compared a single IORT treatment during lumpectomy using a low‐energy X‐ray system (INTRABEAM; Carl Zeiss Surgical) and postoperative CF‐WBI for early breast cancer. In total, 2298 women aged 45 years and older with stage T1‐2b, cN0‐N1, breast cancer eligible for breast conservation were randomized before lumpectomy to either IORT or CF‐WBI with EBRT. If postoperative histopathology found unsuspected higher risk factors in the IORT arm (around 20% of patients), the patients received WBI. The investigators called this “risk adapted IORT.”

Noninferiority was judged with a margin of 2.5% for the absolute difference in IBTR at 5 years between the two arms and long‐term survival outcomes. In total, 1140 patients were randomized to IORT and 1158 to CF‐WBI. At 5 years, the IBTR was 2.11% for IORT and 0.95% for CF‐WBI (difference 1.16%, 90% CI 0.32–1.99). With median follow‐up of 8.6 years, the IBTR was 3.2% for IORT and 1.2% for CF‐WBI. No statistically significant difference was found for both disease‐specific survival and OS.

There was some controversy on this trial regarding its design, since about 20% TARGIT‐IORT patients also received WBI, and the statistical analyses (the use of 90% CI and the choice of noninferiority margin at 5 years). Even with the patient crossover, the IBTR for the IORT arm was higher at both 5 years and 8.6 years. One potential reason is the lack of adequate dose at 1 cm from the lumpectomy cavity surface (5–7 Gy).

#### 2.4.6 ELIOT trial^39^, ^50^

ELIOT stands for “electron intraoperative radiotherapy.” In this randomized, phase III equivalence trial, APBI delivering 21 Gy with the use of an intraoperative electron beam was compared with 50 Gy CF‐WBI with a 10 Gy boost. The electron beam was delivered from a single direction with a chosen cone. Equivalency was judged by 5‐year IBTR for the ELIOT arm not exceeding 2.5 times the presumed 5‐year IBTR of 3% for the CF‐WBI arm. OS was the secondary endpoint.

A total of 1305 women with tumor size <25 mm and clinically negative axillary lymph nodes were randomly assigned (1:1) to either CF‐WBI or ELIOT. At 5 years, the IBTR rates were 4.2% (95% CI 2.8–5.9) with ELIOT and 0.5% (95% CI 0.1–1.3) with CF‐WBI. After a median follow‐up of 12.4 years, the cumulative 10‐year risk of IBTR was 8.1% (6.1–10.3) in the ELIOT group and 1.1% (0.5–2.2) in the CF‐WBI group. No statistical differences were found in OS between the two groups. It is suspected that the higher IBTR in the ELIOT group is associated with geometric misses and inadequate dose coverage of the entire CTV.

#### 2.4.7 Discussions on APBI

These APBI trials used different technologies and dose/fractionation schemes. Aside from the two IORT trials, the use of EBRT and interstitial brachytherapy in the other four trials all showed comparable IBTR to WBI after long‐term follow‐up. Although the NSABP B39 trial^28^ did not reach noninferiority, the difference in 10‐year cumulative incidence of IBTR between APBI and WBI was only 0.7%, while the RAPID trial,^44^ using 3D‐CRT for APBI, resulted in consistently worse cosmetic outcome at 3 and 5 years compared to WBI. The University of Florence trial^47^, ^48^ using IMRT reported that APBI resulted in improved acute toxicity (*p* = 0.0001), late toxicity (*p* = 0.004), and cosmetic outcome (*p* = 0.045).^47^ Additionally, there was no significant difference in terms of IBTR between the WBI and APBI groups. In a separate analysis of patients aged 70 and older, the APBI group had significantly better results in terms of acute skin toxicity, considering both any grade and grade 2 or higher.^48^

It should be noted that the RAPID trial and the EBRT dose used in NSABP B39 and the University of Florence trial used different dose and fractionation schemes—38.5 Gy in 10 fractions twice daily over 5–8 days (RAPID, NSABP B39) and 30.0 Gy in five fractions once daily over 5 days (Florence)—so differences in outcome may not be entirely due to delivery techniques. It has been suggested that the biologically equivalent dose corresponding to 38.5 Gy in 10 fractions delivered twice daily may be too high and the 6‐h interval between the two treatments may not be long enough to allow sufficient normal tissue repair, thus both may have contributed to the poor cosmesis.^44^ In light of the FAST FORWARD result, both the RAPID dose and the Florence dose could be lowered.

A drawback of external beam APBI is that a larger volume of tissue is irradiated than with techniques such as interstitial or intracavitary brachytherapy. This is because of the external origin of the radiation and the need to account for uncertainties in the target location via typically large margins for error. A margin of 10 mm around the entire CTV is typical for 3D‐CRT and IMRT‐based external beam APBI. Within the clinical target and this margin, tissue receives the full prescription dose. Hepel et al.^51^ clearly illustrated the dose–volume effect on late toxicity in APBI using EBRT. For example, the volume receiving 50% of the prescription dose (V50) greater than 40% of the breast volume was associated with high incidence of late subcutaneous fibrosis and poor cosmetic outcome. Brachytherapy does not suffer from this problem because the sources of radiation are fixed relative to the targeted volume of tissue. The need to minimize geometric uncertainty and the required planning target volume (PTV) margin makes stereotactic body radiotherapy a natural direction. A comprehensive review of APBI trials is given by Hikey et al.^52^

## 3 STEREOTACTIC BODY RADIOTHERAPY

After more than two decades of clinical research, APBI is considered to be an efficient and safe adjuvant treatment for low‐risk breast cancer.^52^ While the beam delivery technique characterizing IMRT addresses the issue of conforming dose to an irregular volume and maintaining dose homogeneity, it does not address issues of spatial accuracy in targeting the desired treatment volume. Achievable spatial accuracy directly dictates the geometric margin that must be built in via the PTV expansion. A margin of 1–1.5 cm, on top of the clinical margin for microscopic disease spread, is typically used for EBRT‐based PBI, which can represent a large annulus of normal tissue, especially for larger target volumes.

To further reduce the volume of normal breast and surrounding organs, such as the lungs and heart, receiving large doses of radiation, efforts were made on the application of SRS techniques commonly used to treat brain tumors and other disease conditions to the breast.

A hallmark of SBRT is high doses per fraction. SBRT for early‐stage non‐small‐cell lung cancer is now common and typically uses 12–20 Gy per fraction. Whereas conventionally fractionated radiotherapy is associated with local control rates of less than 50%, lung SBRT has shown control rates of approximate 90%.^53^ There has been an increased interest in applying SBRT in breast cancer.

### 3.1 Current SRS devices used for APBI

#### 3.1.1 CyberKnife

The CyberKnife™ system (Accuray) has been used in several PBI trials as a mode of stereotactic targeting. This device actively tracks motion in real time using implanted fiducial markers (https://www.accuray.com/cyberknife/). For breast cancer treatment, this includes compensation for regular respiratory motion. This technique allows a reduction in or elimination of the margin used for geometric uncertainty. The use of the CyberKnife for APBI, with very small or even no additional margin for uncertainty, has shown encouraging results in terms of cosmesis and other outcomes with highly accelerated treatment regimens.^54^ Robotic SBRT has also been used for three‐fraction preoperative treatment, where target volumes are smaller than in the postoperative setting.^55^

#### 3.1.2 GammaPod

The GammaPod™ system (Xcision Medical Systems) is another device designed specifically for stereotactic radiotherapy of breast cancer.^56^, ^57^ The GammaPod achieves submillimeter accuracy by immobilization of the breast in the prone position and stereotactic localization. The GammaPod is designed to minimize the dose to tissue outside of the intended partial breast target volume, thus potentially avoiding the high incidence of poor cosmetic outcomes observed after EBRT‐based APBI treatment. Design features include a rotating beam geometry that creates a focused “ball” of dose with either 1.5 or 2.5 cm diameter. Each dose ball includes a very rapid fall‐off away from its edge. Driving dose balls in an optimized trajectory within a target volume effectively “paints” the target with dose until the prescription dose level and coverage are reached.

The further design is a vacuum‐assisted breast immobilization cup that maintains the target at a consistent location relative to the cup's stereotactic coordinate system between the time of treatment planning and treatment delivery. The cup also minimizes geometric uncertainty during treatment. Embedded in the outer cup is a fiducial marker acting as the stereotactic frame that can be seen on CT scans used for planning, and it effectively marries the coordinate system used by the treatment planning system to the coordinate system used by the treatment delivery device. This is similar to the Gamma Knife, as both use the principle of immobilization along with stereotactic localization to minimize geometric uncertainties. A low uncertainty in target position means that only a small inbuilt margin for error is required in the PTV.^58^ Use of a smaller target volume inherently reduces the volume of normal tissue irradiated, independent of the beam geometry. The GammaPod is also similar to the Gamma Knife in the use of a large number of convergent cobalt‐60 beams as the means of focusing radiation. Such geometric focusing makes the radiation dose to fall‐off rapidly outside the target volume.^59^, ^60^, ^61^, ^62^, ^63^

### 3.2 Postoperative SBRT

The Swedish Medical Center and Winthrop University jointly conducted a study where 46 patients were postoperatively treated using CyberKnife SBRT.^64^ The Swedish Medical Center used total doses ranging from 25 to 36 Gy in five to 10 fractions (the majority being 10 fractions, twice daily) and Winthrop University used 30 Gy in five daily fractions. Gold fiducial markers were placed near the lumpectomy cavity and patients were positioned with a thermoplastic cast across the chest to stabilize the breast tissue (but note not preventing breathing motion). The PTV margin of only 2 mm (on top of a 1.5‐cm CTV expansion around the lumpectomy cavity) was used due to confidence in the targeting strategy. Cosmetic results were good or excellent in all patients. Note that the use of a 2‐mm PTV margin represents a large reduction in treated volume compared to the 10‐mm margins commonly used in nonstereotactic approaches.

The University of Texas Southwestern (UTSW) Medical Center used CyberKnife SBRT to treat 75 patients with doses from 30 to 40 Gy in five fractions. In most cases, treatment was administered every other day to allow adequate recovery time from the relatively large fractions.^54^ In this trial, no PTV margin was used on top of the 1.5‐mm CTV expansion in a strategy to minimize total volume treated to the prescription dose. Physicians and patients, respectively, scored cosmesis as excellent or good in 100% and 95% of patients at 24 months post treatment. No recurrences were seen.

Georgetown University Hospital treated 10 patients using CyberKnife SBRT to a total dose of 30 Gy in five fractions.^65^ A 5‐mm PTV margin was used. All patients had excellent or good cosmetic outcomes.

The University of Maryland and the UTSW Medical Center are two early adopters of the GammaPod system. They have jointly conducted a trial that gives the tumor bed a single fraction boost dose of 8 Gy as a replacement of the traditional 16 Gy boost in eight fractions.^66^ WBI was delivered mostly with 40 Gy in 15 fractions. As of June 2022, a total of 120 patients were enrolled in these two institutions. At the completion of the entire EBRT, there were 71 grade 1 acute toxicity events, eight grade 2 events, and no grade 3 or higher toxicity. These side effects were not more than commonly seen in WBI. With a median follow‐up of 20 months, there was no IBTR.

Since early 2019, the University of Maryland and the UTSW Medical Center have also jointly conducted an APBI trial using the GammaPod system to deliver 6–8 Gy per fraction for five daily fractions.^67^ As of June 2022, a total of 176 patients had been treated in these two institutions. Acute toxicity was mostly grade 1 with 111 events, with 10 grade 2 events and no grade 3 events reported. With a median follow‐up of 18 months, there were no residual skin changes or remarkable subcutaneous fibrosis. Cosmesis was all excellent.

The Ospedale Santa Maria della Misericordia in Udine, Italy is conducting a single fraction APBI trial delivering a single 17.5 Gy dose to the postoperative cavity plus a 1.0 cm margin using the GammaPod system. As of June 2022, 41 patients had been treated. There were seven grade 1 acute toxicity events observed and no grade 2 or higher acute toxicity was observed (private communication).

These SBRT‐based PBI studies consistently reported outcomes with low toxicity, including good or excellent cosmesis. This contrasts with results using more traditional EBRT delivery techniques, such as those used in the RAPID trial (3D‐CRT).

### 3.3 Preoperative SBRT

Targeting the intact tumor can allow a substantial decrease in treatment volume compared with targeting the postoperative lumpectomy cavity wall or seroma volume. For example, a University of Maryland study examined pre‐ and postlumpectomy PTV for 41 patients and found that the median volumes were 93 and 250 cm^3^, respectively.^68^ Preoperative radiation treatment therefore potentially improves cosmetic outcomes and reduces toxicity. There is also less interobserver variability with preoperative tumor delineation than postoperative lumpectomy delineation because of the clarity of the intact tumor on imaging studies.^69^ Preoperative treatment may be used along with neoadjuvant chemotherapy (NACT) to shrink the tumor prior to surgery. Patients initially ineligible for BCT because of a large tumor diameter may then become eligible.

The University Hospital of Nice conducted a preoperative SBRT study with 25 patients using the CyberKnife system to deliver doses of 19.5–31.5 Gy in three fractions concurrent with neoadjuvant chemotherapy.^55^ Breast‐conserving surgery was able to be performed in 92% of patients who were not initially eligible for BCT. A single case of dose‐limiting skin toxicity was reported. There were no local recurrences. This study showed the safety of doses up to 31.5 Gy in three fractions with low toxicity and good response rates.

Duke University used a linear accelerator‐based stereotactic technique to deliver doses of 15, 18, or 21 Gy to 32 patients in a single fraction.^70^ Most were treated in the prone position (thus minimizing respiratory motion) and with a 3‐mm PTV margin. The biopsy clip in the tumor was used as a fiducial marker during image guided setup. Cosmesis was excellent or good in 14 of 15 patient‐reported outcomes and 15 of 15 physician‐reported outcomes.

The Netherlands Cancer Institute initiated another preoperative PBI trial, with Institute Gustave Roussy in France, the Karolinska Institute in Sweden, and University Medical Centre Utrecht also participating in it. Ten fractions of 4 Gy were delivered using 3D‐CRT, IMRT, or volumetric modulated arc therapy (VMAT). Image guidance using cone beam CT or portal imaging was used to maintain setup accuracy and a PTV margin of 5 mm was used. The primary endpoints were local recurrence, breast fibrosis, and cosmetic outcome. Results for 70 patients have been published.^71^ After 1, 2, and 3 years of follow‐up, the proportions of patients with a good to excellent cosmetic outcome were 89%, 88%, and 100%, respectively; at 1, 2, and 3 years of follow‐up, 89%, 98%, and 100% of patients had no or mild induration fibrosis and two patients developed a local recurrence.

### 3.4 Stereotactic radiosurgery

There are several currently‐accruing breast SRS trials in the USA, Canada, and Europe. Of note is the ABLATIVE trial carried out by the University Medical Center Utrecht in the Netherlands.^72^ A total of 25 consecutive patients are being treated using VMAT with a single ablative radiotherapy dose of 20 Gy to the tumor and 15 Gy to the tumor bed. An “ablative” dose is, by definition, large enough to kill all cells within the targeted volume. The key is therefore to be able to minimize this volume while still including all tumor cells. MRI guidance is used to maintain spatial accuracy, allowing a PTV margin of 3 mm. The purpose of the study is to evaluate the feasibility of single‐dose radiotherapy as definitive (primary) treatment for early‐stage breast cancer. The study tests the hypothesis that definitive radiation treatment can take the place of surgery plus adjuvant radiotherapy (WBI or PBI) for the low‐risk patient group currently eligible for BCT. Breast‐conserving surgery will be performed at 6 months after radiotherapy, with pathological complete response (pCR) the primary endpoint, assessed by microscopic evaluation of the excision specimen.

Another breast cancer SRS trial, SPORT‐DS (Single Pre‐Operative Radiation Therapy—With Delayed Surgery) is ongoing at the Maisonneuve‐Rosemont Hospital, Montreal.^73^ Eligibility includes tumor size ≤2 cm, ER positive, human epidermal growth factor receptor 2 (Her2) negative, and age 65 years or older. The PTV is defined as 1 cm expansion from the CTV, which is 1 cm expansion from the gross target volume (GTV). Surgery will be performed at least 3 months after SRS with 20 Gy to the PTV. The primary endpoint is pCR.

Other breast cancer SRS trials include those at Johns Hopkins^74^ and Duke University.^75^ The Johns Hopkins study intends to enroll 40 patients while Duke intends to enroll 100 patients, both in a single arm, and deliver a single dose of 21 Gy preoperatively to the GTV plus a small margin with linear accelerator in prone position. The primary endpoint is cosmesis with pCR as the secondary end point.

Three of the institutions with the GammaPod system are currently enrolling patients to three separate breast cancer SRS studies. These include the Dose Escalation Study of Single Fraction Early Stage Breast Cancer at UTSW.^76^ A single dose of 30, 34, and 38 Gy will be delivered to the GTV plus a 5‐mm margin preoperatively. With 30 Gy as an initial dose, 15 patients are enrolled in each dose level. If the treatment is well tolerated, the study will proceed to the next dose level, judged by acute toxicity grades, until the maximum tolerable dose (MTD) is reached or 38 Gy. Surgery will be performed at least 1 month from SRS. The primary end point is MTD and cosmesis, with pCR being the secondary endpoint. By the end of June 2022, 25 patients had received a single fraction of 30–34 Gy treatments.

The SRS trial at the University of Maryland is nearly identical to that of UTSW in every aspect except the dose levels being studied. A lower single dose of 21, 24, 27, and 30 Gy will be delivered.^77^

As of the end of July 2022, the Ospedale Santa Maria della Misericordia in Udine, Italy had also enrolled 33 patients with early‐stage breast cancer to a single‐arm definitive SRS study. A single dose of 30–33 Gy is delivered to the GTV plus a margin of 3–5 mm using the GammaPod system. Patient eligibility criteria include grade 1 to 2b (tumor less than 3 cm), ER positive, and Her2 negative. Tamoxifen will be given after SRS treatment, and surgery will be performed at 3–6 months following SRS treatment. The primary end point is pathologic complete response. The secondary endpoints are acute and late toxicity, and cosmesis. The treatment is well‐tolerated with no grade 2 or higher acute toxicity events. (private communication).

SRS used as the definitive treatment for early‐stage breast cancer represents a drastic shift in the management of breast cancer. If proven effective, it will not only shorten local/regional therapies from months to a single noninvasive treatment, it will also eliminate the delay of systemic therapies. As with SBRT of non‐small‐cell lung cancer, the final verdict will come from randomized phase III clinical trials. Therefore, years or even decades of clinical research may be needed for SRS to become an accepted treatment option for early‐stage breast cancer.

## 4 CONCLUSION

Over the last three decades, the increased awareness of and advances in breast cancer screening have led to the majority of breast cancers being diagnosed at an early stage. With the introduction of tamoxifen, trastuzumab, aromatase inhibitors, and other targeted agents, the role of radiation therapy in managing early‐stage breast cancer has been challenged. However, in all of the phase III trials, the addition of radiation consistently translated to substantial reductions of IBTR. As an easier and gentler local/regional therapy, radiotherapy in particular has been a driving factor of the progression in the management of breast cancer. During this period, breast cancer treatment has progressed from mastectomy to breast conservation therapy, hypofractionated WBI, APBI, and SBRT. Hopefully, SRS can show its potential of replacing both surgery and postoperative irradiation in the next decade, drastically improving the quality of life for millions of women inflicted with this disease.

## CONFLICT OF INTEREST

The author is the founder, CEO, and shareholder of Xcision Medical Systems, LLC., the manufacturer of the GammaPod system for stereotactic radiation therapy of breast cancer. His financial interest in the company may present a conflict of interest in the contents of this review.
